# Integrative omics to detect bacteremia in patients with febrile neutropenia

**DOI:** 10.1371/journal.pone.0197049

**Published:** 2018-05-16

**Authors:** Rachel S. Kelly, Jessica Lasky-Su, Sai-Ching J. Yeung, Richard M. Stone, Jeffrey M. Caterino, Sean C. Hagan, Gary H. Lyman, Lindsey R. Baden, Brett E. Glotzbecker, Christopher J. Coyne, Christopher W. Baugh, Daniel J. Pallin

**Affiliations:** 1 Channing Division of Network Medicine, Brigham and Women’s Hospital, Boston, MA, United States of America; 2 Harvard Medical School, Boston, MA, United States of America; 3 Department of Emergency Medicine, The University of Texas MD Anderson Cancer Center, Houston, TX, United States of America; 4 Dana Farber Cancer Institute, Boston, MA, United States of America; 5 Ohio State University Medical School, Wexner Medical Center Department of Emergency Medicine, Columbus, OH, United States of America; 6 Department of Emergency Medicine, Brigham and Women’s Hospital, Boston, MA, United States of America; 7 Fred Hutchinson Cancer Research Center, Seattle, WA, United States of America; 8 School of Medicine, University of Washington, Seattle, WA, United States of America; 9 University of California, San Diego, School of Medicine and Department of Emergency Medicine, San Diego, CA, United States of America; National Research Council of Italy, ITALY

## Abstract

**Background:**

Cancer chemotherapy-associated febrile neutropenia (FN) is a common condition that is deadly when bacteremia is present. Detection of bacteremia depends on culture, which takes days, and no accurate predictive tools applicable to the initial evaluation are available. We utilized metabolomics and transcriptomics to develop multivariable predictors of bacteremia among FN patients.

**Methods:**

We classified emergency department patients with FN and no apparent infection at presentation as bacteremic (cases) or not (controls), according to blood culture results. We assessed relative metabolite abundance in plasma, and relative expression of 2,560 immunology and cancer-related genes in whole blood. We used logistic regression to identify multivariable predictors of bacteremia, and report test characteristics of the derived predictors.

**Results:**

For metabolomics, 14 bacteremic cases and 25 non-bacteremic controls were available for analysis; for transcriptomics we had 7 and 22 respectively. A 5-predictor metabolomic model had an area under the receiver operating characteristic curve of 0.991 (95%CI: 0.972,1.000), 100% sensitivity, and 96% specificity for identifying bacteremia. Pregnenolone steroids were more abundant in cases and carnitine metabolites were more abundant in controls. A 3-predictor gene expression model had corresponding results of 0.961 (95%CI: 0.896,1.000), 100%, and 86%. Genes involved in innate immunity were differentially expressed.

**Conclusions:**

Classifiers derived from metabolomic and gene expression data hold promise as objective and accurate predictors of bacteremia among FN patients without apparent infection at presentation, and can provide insights into the underlying biology. Our findings should be considered illustrative, but may lay the groundwork for future biomarker development.

## Introduction

Bacteremia due to chemotherapy-associated febrile neutropenia (FN) is one of the most deadly oncologic emergencies, with mortality rates of up to 50%.[1, 2] Therefore, presumptive broad-spectrum antibiotic treatment is recommended for all FN patients within 1 hour of onset of symptoms, and the vast majority are admitted to the hospital.[3–6] However, fewer than 25% of FN patients are found to have bacteremia.[1, 3, 4] Antibiotic treatment carries risks including *C*. *difficile* colitis, selection of drug-resistant strains, drug toxicity, allergic reactions, and drug-drug interactions, while hospitalization confers risks of acquisition of nosocomial pathogens, exposure to medical errors, catheter-associated infections, thromboembolism, and financial burdens.[3, 4, 7–9]

Currently, detection of bacteremia depends on culture, which rules out bacteremia only after several days; too late to inform initial decisions regarding hospitalization and therapy. Guidelines recommend that initial treatment decisions be based on clinical evaluation including the Multinational Association for Supportive Care in Cancer (MASCC) score.[4, 10] However, this score was not designed to detect bacteremia, and is insufficiently accurate even for its intended use, prediction of safe discharge, with a negative predictive value for complications of only 83%.[11] Consequently, many clinicians do not rely on it, admitting all FN patients by default.[5, 6] A newer score, the Clinical Index of Stable Febrile Neutropenia, is also inadequate, with a 9.1% rate of bacteremia in the low-risk group.[12] Similarly, PCR for bacterial DNA, and measurement of host markers such as procalcitonin, lack sufficient sensitivity.[13–15] Objective tests are needed to detect bacteremia during the initial evaluation, so that patient-specific management strategies can be employed.

High throughput ‘omics’ profiling is a powerful tool for the discovery of biomarkers for various conditions, including infectious diseases.[16] Metabolomics, which provides an integrated profile of biological status, reflecting the “net results” of genetic, transcriptomic, proteomic, and, environmental interactions, represents a particularly powerful research tool.[17] Differentially-abundant metabolites may be utilized as biomarkers to discriminate between those with and without bacteremia.[18, 19] By integrating gene-expression data with metabolomic data, we can identify changes in upstream regulators of the metabolites of interest, strengthening pathway analyses and facilitating deeper understanding of underlying biology.[20, 21]

The aim of this study, was to develop methods for the development of omic biomarkers that can accurately identify bacteremia among FN patients without apparent infection. Eventually, we hope to build upon the most promising results from this project to develop a multivariable biomarker that can be advanced into a test for routine clinical use, whilst informing on underlying biology.

## Methods

### Study population

Between October 2015 and October 2016, we recruited patients with fever (defined as a single oral temperature ≥38.3°C or an oral temperature ≥38°C sustained over a 1-hour period)[4, 10] and FN (defined as <1000 neutrophils/μL_2_), from the Brigham and Women’s Hospital emergency department (receiving emergency department for the Dana Farber Cancer Institute). All patients had detailed clinical evaluations including history, physical exam, chest X-ray, urinalysis, and, blood and urine cultures. Information on antibiotic use in the 24 hours prior to blood collection was also recorded. Patients presenting with focal bacterial infection (pneumonia, skin infection, urinary tract infection, intra-abdominal infection) or evidence of sepsis in the judgment of two investigators were excluded, as the goal of this study was to find an indicator of bacteremia in patients without initially-detectable bacterial infection. Trained research assistants screened for eligible patients between the hours of 7am and 11pm daily. Once subjects were identified, they collected two blood samples (in PAXgene RNA preservation tubes and lithium heparin tubes) and clinical data. Two investigators then independently categorized each subject, relying upon the diagnostic investigation conducted as part of routine care and the recommendations of the Infectious Diseases Society of America and the American Society of Clinical Oncology.[4, 10, 22] The investigators placed each subject into one of three categories: (i) No evidence of focal infection or sepsis at presentation and ultimately found to have been bacteremic (cases), (ii) No evidence of focal infection or sepsis at presentation and ultimately found not to have been bacteremic (controls), or (iii) Evidence of focal infection or sepsis at presentation (excluded),. The Partners Health Care Human Subjects Research Committee approved this study, and all participants provided written informed consent.

### Metabolomic and gene expression profiling

Mass spectrometry-based metabolomic profiling was performed on plasma samples by Metabolon, Inc. (Durham, NC), as described previously [23, 24] Briefly, the global biochemical profiling analysis was composed of four unique arms covering a broad range of the metabolome; (i) reverse-phase chromatography positive ionization methods optimized for hydrophilic compounds (LC–MS Pos Polar) and (ii) hydrophobic compounds (LC–MS Pos Lipid); (iii) reverse-phase chromatography with negative ionization conditions (LC–MS Neg), and (iv) a HILIC chromatography method coupled to negative (LC–MS Polar). Metabolites were annotated based on an iterative process of matching on mass to charge ratio, retention time and spectral fragmentation signature, followed by manual curation to confirm biochemical identification. Further details are provided in **S1 Methods**.

RNAseq could not be used in this population due to the paucity of white blood cells. We used EdgeSeq from HTG Molecular, Inc (Tucson, AZ).[25] to quantify the expression of 2,560 genes relevant to cancer and immunology. This technology does not require an RNA isolation step. The panel of genes was chosen by an iterative process of literature review and key opinion leader feedback, and included 24 gene groups and pathways.[26]. Analysis was performed as described previously [25, 26]. Briefly, the EdgeSeq assay couples quantitative nuclease protection with next-generation sequencing. After allowing nuclease protection probes (NPPs) to hybridize to their target RNAs, S1 nuclease is added to remove excess unhybridized NPPs and RNA, leaving behind only NPPs hybridized to their target RNAs, resulting in a stoichiometric conversion of target RNA to the NPPs and producing a 1:1 ratio of NPP to RNA. The quantitative nuclease protection steps are automated on the EdgeSeq processor, followed by PCR to add sequencing adaptors and tags. The labeled samples are pooled, cleaned, and sequenced on a next-generation sequencing platform using standard protocols. The resulting data are processed and reported by EdgeSeq parsing software. Further details are provided in **S1 Methods**.

### Quality control and data processing

#### Metabolomics

We used a previously published [27] QC and processing pipeline to clean the metabolomic dataset. We excluded metabolites with zero abundance in all samples, then imputed all remaining missing observations with half the lowest detected value for that metabolite. We considered metabolites with zero variance uninformative and excluded them. We then *pareto* scaled and log-transformed the data.

#### Gene expression

We performed data processing and normalization according to EdgeSeq manufacturer’s standards.[25, 26] Data were transferred from the Illumina MiSeq sequencer as demultiplexed FASTQ files, with one file per original well of the 96-well sample plate. The HTG EdgeSeq Parser was used to align the FASTQ files to the probe list to collate the data, which were then median-normalized.[28]

### Statistical analysis

#### Prediction

We used unsupervised principal components analysis (PCA) to assess the ability of the metabolome and gene expression data to discriminate cases (with bacteremia) from controls (without bacteremia). We further interrogated these plots to determine if other clinical factors may be driving the metabolomic profiles. We then used supervised partial least squares discriminant analysis (PLS-DA) to assess predictive accuracy for bacteremia. Next, we attempted to identify metabolomics and genetic predictive profiles using two approaches; (i) We used independent logistic regression models adjusting for age, sex, body mass index (BMI), and, tumor type (solid/liquid) to identify the metabolites most strongly associated with the presence or absence of bacteremia. Differential gene expression analysis was used for the gene expression data. (ii) We employed least absolute shrinkage and selection (LASSO) sparsity-inducing logistic regression to identify more parsimonious metabolomic and genomic predictors. We ran two logistic models; one containing all metabolites, and one containing all genes to identify the subset of metabolites and genes retained in the models. These were then selected as the predictors. We used the lambda that produced the minimum mean cross validated error.[29]

We then created metabolite and gene summary scores based on (i) the first principal component of metabolites/expressed genes identified as differentially abundant in cases vs. controls in the regression models; and (ii) metabolites/genes selected in the LASSO model. We used receiver operating characteristic (ROC) curve analysis to evaluate the predictive ability of these summary scores for bacteremia, and employed the method of DeLong to compare areas under the ROCcurves for the different scores.[30] We determined a cutoff that maximized sensitivity, and calculated specificity at this cutoff. The currently recommended approach to risk stratification is to classify a patient as high-risk if the MASCC score is <21 or if any of the Infectious Diseases Society of America/American Society of Clinical Oncology high risk criteria are met.[3, 4] While this approach to risk stratification was not designed to detect bacteremia, no other method currently exists to classify these patients. Therefore, we compared the accuracy of this classifier as a predictor of bacteremia to the accuracy of our omic predictors of bacteremia.

#### Analysis of underlying biology

We explored metabolic pathways using MetaboAnalyst (http://www.metaboanalyst.ca/), which takes both overrepresentation and pathway topology into account, assigning more weight to metabolites that form key components or ‘hubs’ of specific pathways. For gene expression, we performed gene set enrichment analysis using the g.GOSt tools from the g.profiler package (http://biit.cs.ut.ee/gprofiler/).

In order to combine the findgins from the metabolomics and genetic analysis, we used IMPaLA (Integrated Molecular Pathway Level Analysis; http://impala.molgen.mpg.de/) [31] to identify pathways that were jointly dysregulated at the level of both metabolites and gene expression. IMPaLA performs over-representation analysis considering both genes and metabolites to provide a combined pathway p-value as well as a q-value that accounts for multiple testing.

## Results

### Study population

Metabolomic profiling was performed for 58 subjects who were classified by two investigators with no disagreements. Fourteen (24%) had bacteremia (cases); 25 (43%) had no evidence of bacterial infection (controls); and, 19 (33%) had evidence of a focal infection without bacteremia (excluded from analysis), resulting in a total of 39 analyzed subjects (**Table 1**). Gene expression data were available for only seven of the cases and 21 of the controls, due to logistical issues. A further control had gene expression profiling only. In total seven cases and 22 controls were included in the gene expression analysis.

**Table 1:** Baseline characteristics of the study population.

		Cases (n = 14)		Controls (n = 25)		p-value
Sex	Female	4	28.6%	13	52.0%	0.193
	Male	10	71.4%	12	48.0%	
Age (years)	mean (SD)	55.1 (11.6)		47.0 (16.4)		0.082
BMI	mean (SD)	26.4 (2.7)		25.3 (4.3)		0.310
Tmax (^o^F)	mean (SD)	102.2 (0.8)		101.3 (0.8)		2x10^-3^*
Absolute Neutrophil Count	mean (SD)	0.12 (0.22)		0.35 (0.31)		0.012*
Absolute Lymphocyte Count	mean (SD)	0.21 (0.20)		0.56 (0.51)		0.005*
MASCC risk score	mean (SD)	15.0 (4.3)		18.1 (3.8)		0.032
MASCC high risk	≥21 (low risk)	1	7.1%	8	32.0%	0.120
	<21 (high risk)	13	92.9%	17	68.0%	
ASCO	high	14	100.0%	22	88.0%	0.540
	low	0	0.0%	3	12.0%	
IDSA	high	13	92.9%	20	80.0%	0.391
	low	1	7.1%	5	20.0%	
Tumor type	liquid	12	85.7%	11	44.0%	0.017
	solid	2	14.3%	14	56.0%	
Cancer type^a^	Breast	0	0.0%	3	12.0%	0.441
	Esophageal	0	0.0%	2	8.0%	
	Gynecological	1	7.1%	2	8.0%	
	Hematological	12	85.7%	10	40.0%	
	Lung	0	0.0%	1	4.0%	
	Male reproductive	0	0.0%	2	8.0%	
	Other	1	7.1%	4	16.0%	
	Skin	0	0.0%	1	4.0%	
Antibiotics prior to blood draw^b^	Yes	14	100.0%	19	76.0%	0.071
	No	0	0.0%	6	24.0%	
Organism^c^	Gram-Negative	8	57.1%	-		
	Gram-Positive	5	35.7%	-		
	Both	1	7.1%	-		
Gene Expression Data^d^	Available	7	52.9%	21	84.0%	0.033*

*Indicates a significant difference between cases and controls at a 95% confidence level

Tmax–maximum temperature

MASCC–Multinational Association for Supportive Care in Cancer

ASCO–American Society of Clinical Oncology binary classifier

IDSA–Infectious Diseases Society of America binary classifier

SD–Standard deviation

^a^ Information on cancer type was not available for one control

^b^ The patient received antibiotics in the 24 hours prior to blood draw

^c^ Type of bacteria ultimately identified in the culture samples from bacteremic cases

^d^Transcriptomic analysis was conducted on 7 cases and 22 controls–one of these controls did not have metabolomic profiling available

Cases demonstrated a significantly higher maximum temperature, lower neutrophil and lymphocyte counts, and a lower MASCC score as expected. Patients with both solid and liquid tumors originating from a variety of organs were included. Cases were more likely to have a liquid tumor than controls, but there was no significant difference in tumor site. Antibiotic treatment was initiated prior to sample collection in 85% of subjects; there was no significant difference in the proportion of cases and controls who received antibiotics (**Table 1**).

### Metabolomics

A total of 1,296 metabolites were measured. After exclusion of metabolites that were missing for all subjects and those with no variance across the population, 1,204 metabolites remained for analysis. These included amino acids, carbohydrates, lipids, nucleotides, vitamins, peptides, energy metabolites, and 163 xenobiotics. PCA based on all 1,204 metabolites revealed separation between cases and controls along the first two components which together explained 27% of the variance in the data (**Figure A in S1 File**). To determine if these metabolomic profiles were driven by other clinical factors we also interrogated the PCA plot in terms of tumor type (liquid or solid); tumor site and antibiotic use prior to blood draw. Among the bacteremic cases only, we also explored the bacteria type subsequently identified in the culture (Gram-positive, Gram-negative or both). These plots indicated no clustering based on any of these variables; and therefore provided no evidence that these factors were driving the metabolomic profiles (**Figure B in S1 File**). Regression models confirmed that both PC1 (p = 8.8x10^-4^) and PC2 (p = 0.024) were significantly associated with case status.

**Fig 1:**
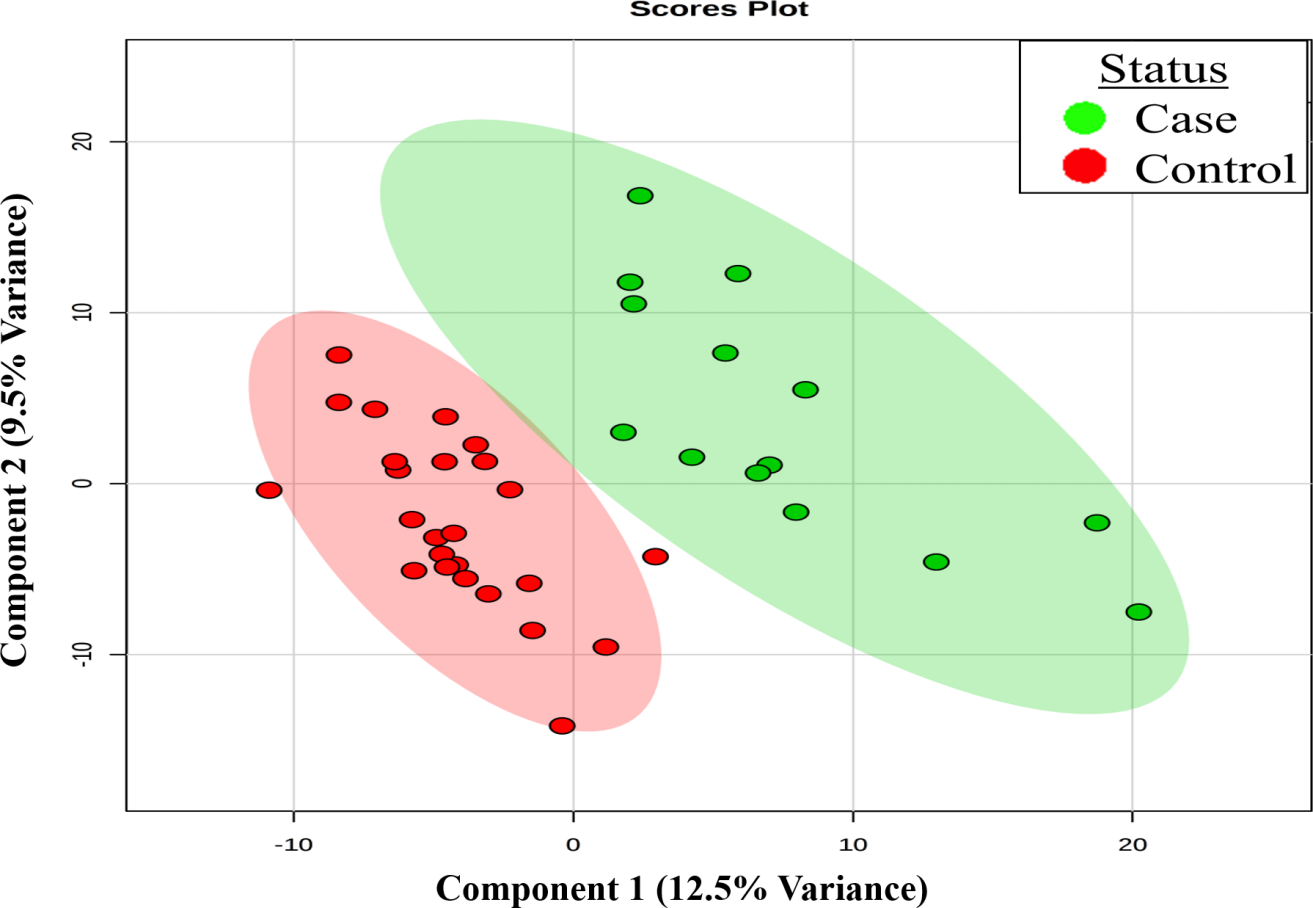
Partial least square discriminant analysis demonstrating metabolomic differences in bacteremia cases (n = 14) and controls (n = 25). The first two components and the corresponding percentage of the total variance in the metabolome explained by these two components are presented.

Partial least squares discriminant analysis (**Fig 1**) suggested that a metabolomic classifier could distinguish between cases and controls, with R^2^ = 0.650, and a cross-validated Q^2^ of 0.410 for the first component. Interrogation of the variable importance in the projection plot (VIP; a measure of the relative importance of each feature in the PLS-DA) identified 17α-hydroxypregnanolone glucuronide, estrone 3-sulfate, 5α-pregnan-3, 20β-diol disulfate and pregn steroid monosulfate (C21H3405S) as the top metabolites driving the discrimination. Carnitines, also had high VIP scores.

**Fig 2:**
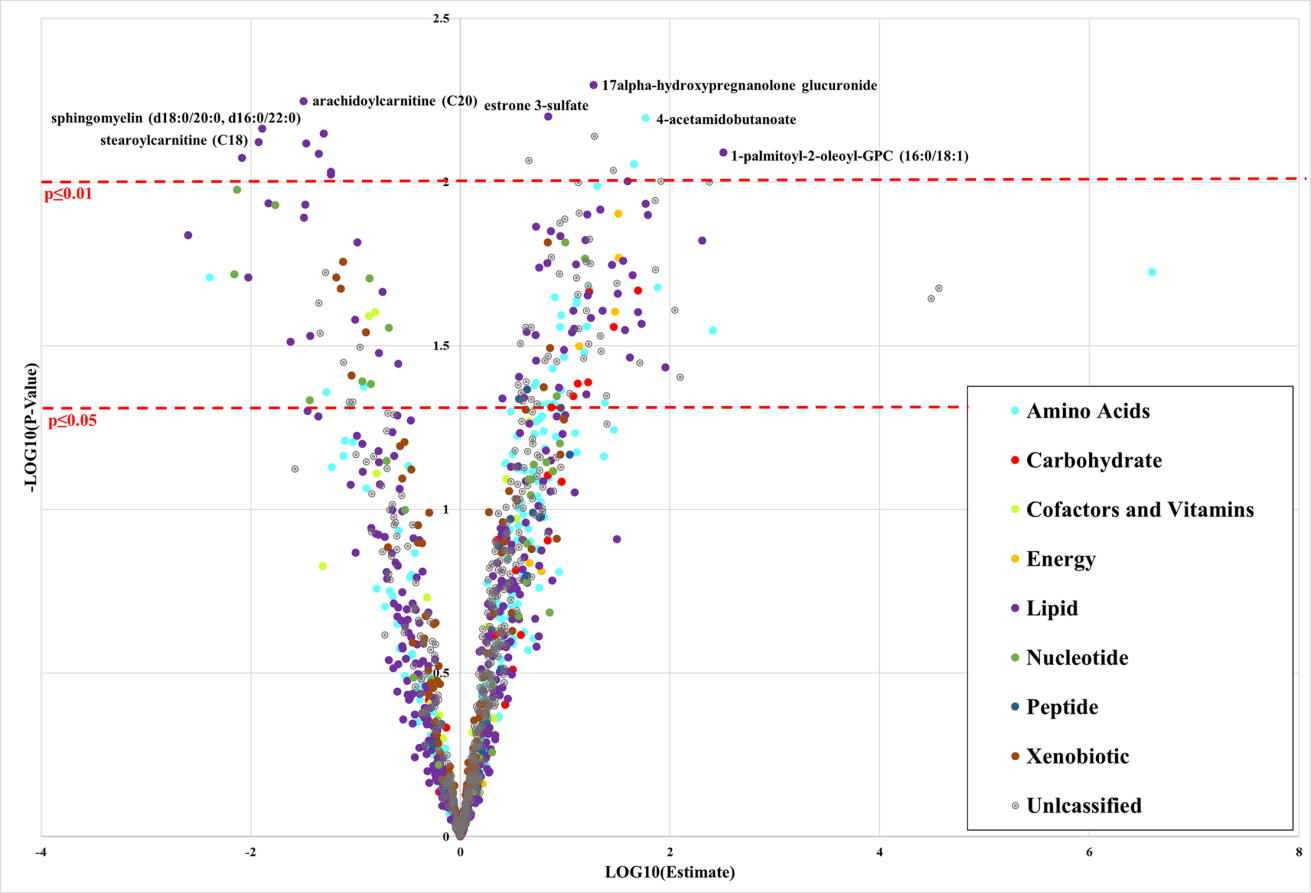
Association between metabolites and bacteremia using a multivariable logistic regression model after adjustment for age, sex, BMI and tumor type (liquid or solid). Metabolites are colored according to their super pathway assignment and top metabolites are named; nominal significance levels of 95% and 99% are indicated with dashed red line; the x axis represents the strength of the association and the y-axis the significance–metabolites to the right of the plot are at higher levels in cases than controls, metabolites to the left are at higher levels among the controls.

Permutation testing revealed that the model was not robust (p = 0.366). Therefore, a refined discriminatory profile was sought by identifying metabolites significantly associated with bacteremia using multivariable logistic regression. After adjustment for age, sex, BMI and, tumor type (liquid or solid), a total of 177 metabolites were significant at p<0.05 and 19 were significant at a p<0.01 (**Fig 2** and **Table A in S1 File**). A majority of the significant metabolites were lipids. We observed upregulation of pregnenolone steroids and downregulation of carnitine metabolites among bacteremia cases. The relative metabolite intensities in cases and controls for the top eight upregulated and top eight downregulated metabolites are shown in **Figure C in S1 File**. Pathway analysis identified six metabolic pathways that were enriched among these significant metabolites: pyrimidine metabolism (p = 0.002), ascorbate and aldarate metabolism (p = 0.003), purine metabolism (p = 0.017), sphingolipid metabolism (p = 0.018), pantothenate metabolism (p = 0.022) and valine, leucine and isoleucine metabolism (p = 0.022).

**Fig 3:**
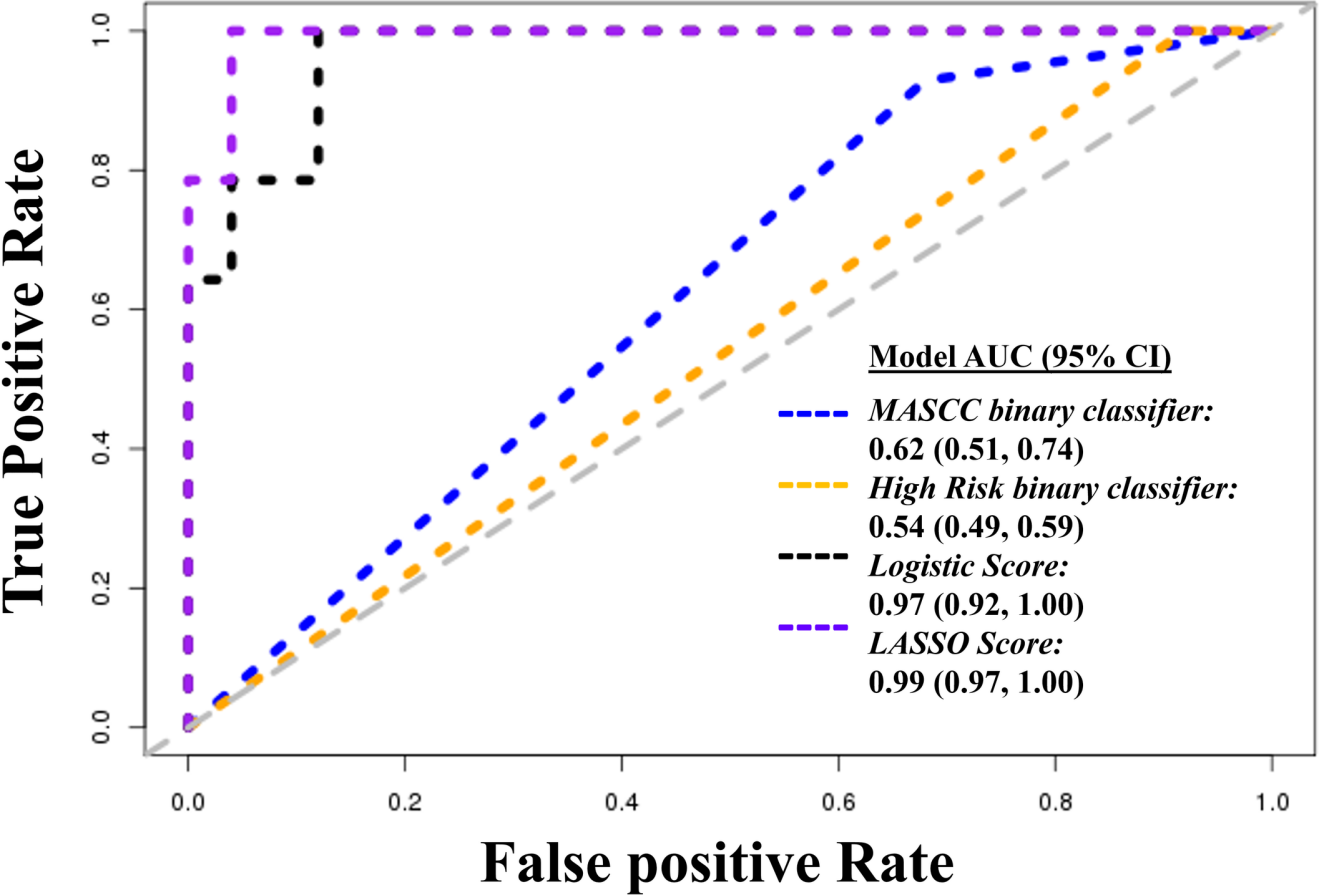
Receiver operating characteristic curves showing the performance of metabolomic (logistic and LASSO) predictors compared to existing clinical (MASCC and high-risk) classifiers. *AUC–Area under the receiver operating characteristic curve*. *MASCC binary classifier-Multinational Association for Supportive Care in Cancer score categorized into <21 (high risk) and ≥21(low risk)*. *High risk binary classifier- defines a patient as high risk if the MASCC score is <21 or if any of the* Infectious *Diseases Society of America*/*American Society of Clinical Oncology high risk criteria are met*. *Logistic score–summary score based on the 137 metabolites associated with bacteremia under a multivariable logistic regression model*. *LASSO score–summary score based on the seven metabolites associated with bacteremia under a penalized LASSO model*.

We generated a summary score based on the first principal component of the 177 metabolites, and used ROC curve analysis to explore the discriminatory ability of this score (**Fig 3**). To identify the most parsimonious model, we then used LASSO regression, and identified a five-metabolite score. The AUCs of the two currently available clinical classifiers; MASCC and MASCC plus an indicator of risk (“high-risk” classifier), were 0.624 (95%CI: 0.508–0.741) and 0.540 (95%CI: 0.486–0.594), respectively. Both metabolite scores significantly out-performed these classifiers with AUCs of 0.969 (95%CI: 0.918–1.000) (*p dif* MASCC classifier = 2.3x10^-8^; *p dif* high risk classifier <2.2x10^-16^) for the standard logistic score and 0.991 (95%CI: 0.972–1.000) (*p dif* MASCC classifier = 8.0x10^-10^; *p dif* high risk classifier <2.2x10^-16^) for the LASSO score. Furthermore, while the sensitivity of the binary MASCC and “high risk” classifiers was high (93% and 100%, respectively), the corresponding specificities were only 32% and 8%. In contrast, at the cutoff required to achieve 100% sensitivity, the specificity of the standard logistic classifier was 88%, and the parsimonious LASSO classifier was 96% (**Table 2**).

**Table 2:** Sensitivity and specificity of the metabolomic and gene expression classifiers compared to existing clinical predictors in terms of area under the curve, sensitivity and specificity.

Classifier	AUC	95%CI	Sensitivity	Specificity
**Metabolomic Data**				
MASSC ≤21	0.624	(0.508, 0.741)	93%	32%
High Risk Classification	0.54	(0.486, 0.594)	100%	8%
Logistic Score optimal cutoff	0.969	(0.918, 1.000)	100%	88%
LASSO Score optimal cutoff	0.991	(0.972, 1.000)	100%	96%
**Gene Expression Data**				
MASSC ≤21	0.61	(0.437, 0.784)	89%	36%
High Risk Classification	0.546	(0.484, 0.607)	100%	9%
Logistic Score optimal cutoff	0.974	(0.926 1.000)	100%	86%
LASSO Score optimal cutoff	0.961	(0.896, 1.000)	100%	86%

AUC–Area under the receiver operating characteristic curve

MASCC binary classifier–Multinational Association for Supportive Care in Cancer score categorized into <21 (high risk) and ≥21(low risk)

High risk binary classifier–defines a patient as high-risk if the MASCC score is <21 or if any of the Infectious Diseases Society of America/American Society of Clinical Oncology high risk criteria are met

Logistic and LASSO score cutoffs were chosen to obtain 100% sensitivity

#### Sensitivity analyses

A sensitivity analysis additionally adjusting for antibiotic use in the 24 hours prior to blood draw identified 118 significant metabolites; 112 (95%) of which were also among the 177 identified in the original analyses. We ran a further sensitivity analysis adjusting for the difference between absolute neutrophil and absolute leukocyte count. Again, of the 169 metabolites that retained significance in this model, 159 (94%) were among the original 177 metabolites., demonstrating the robustness of these findings.

Similarly, we wanted to determine whether the type of bacteria (Gram-negative or Gram-positive) responsible for the bacteremia influenced the results. Although the numbers were too small (n = 5 cases) for the model to converge when considering only at Gram-positive bacteria, we identified 129 metabolites significantly associated with Gram-negative bacteria (n = 8 cases). Ninety-nine (77%) of these were also among the 177 metabolites; including many of the most significant hits such as the carnitines and pregnenolone steroids. Furthermore, when comparing the relative levels of these top metabolites in controls versus cases stratified by Gram-status, both the Gram-negative and Gram positive bacteria cases were distinct from the controls (**Figure D in S1 File**). This again suggests that case-control status rather than bacteria type among the cases was the biggest driver of the differential metabolite abundance.

### Gene expression profiling

**Fig 4:**
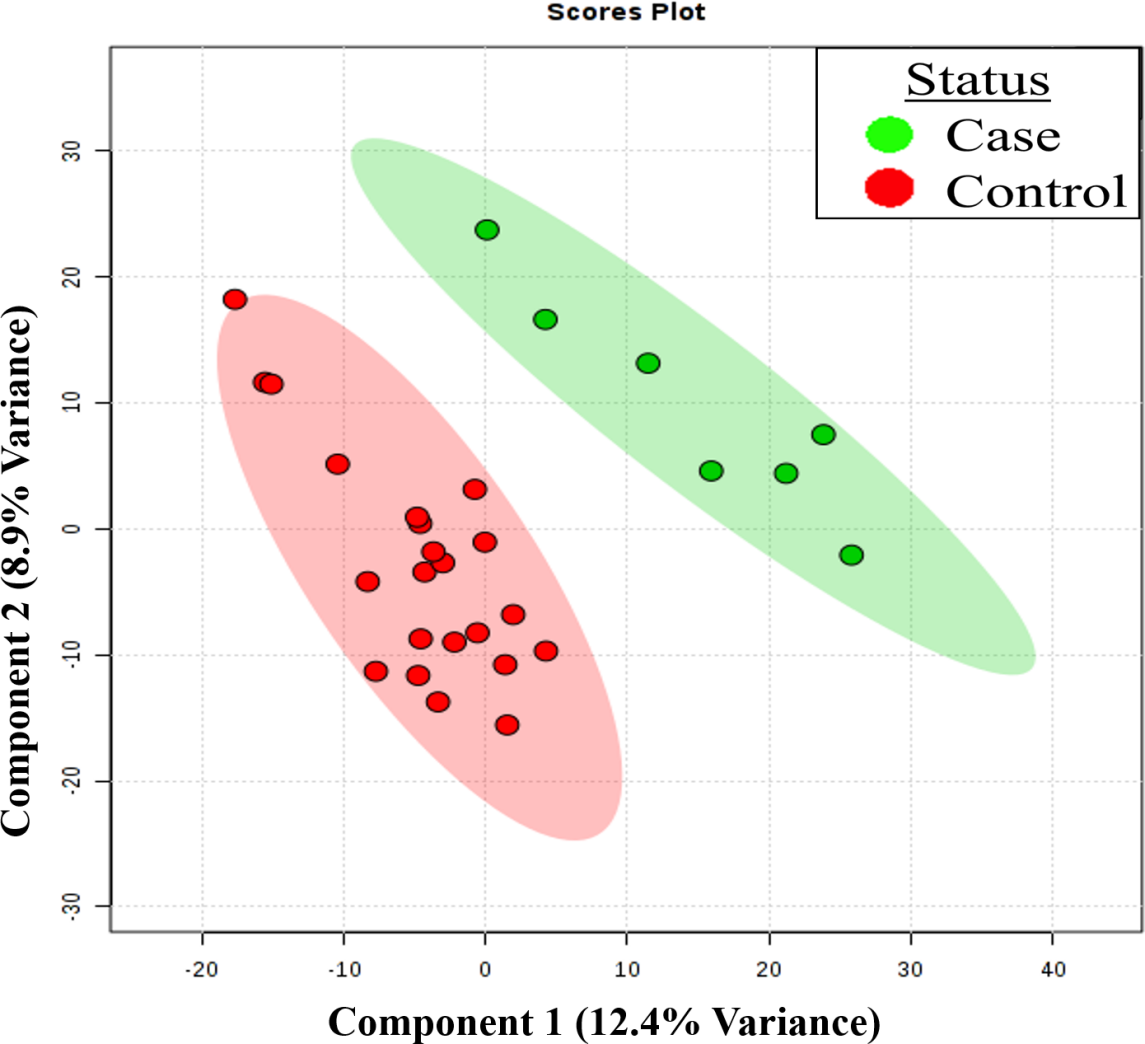
Partial least square discriminant analysis demonstrating differential gene expression in bacteremia cases (n = 9) and controls (n = 21). Gene expression profiles based on 2560 genes quantified using EdgeSeq; the first two components and the corresponding percentage of variances in the gene expression profile explained by these two components are presented.

**Fig 5:**
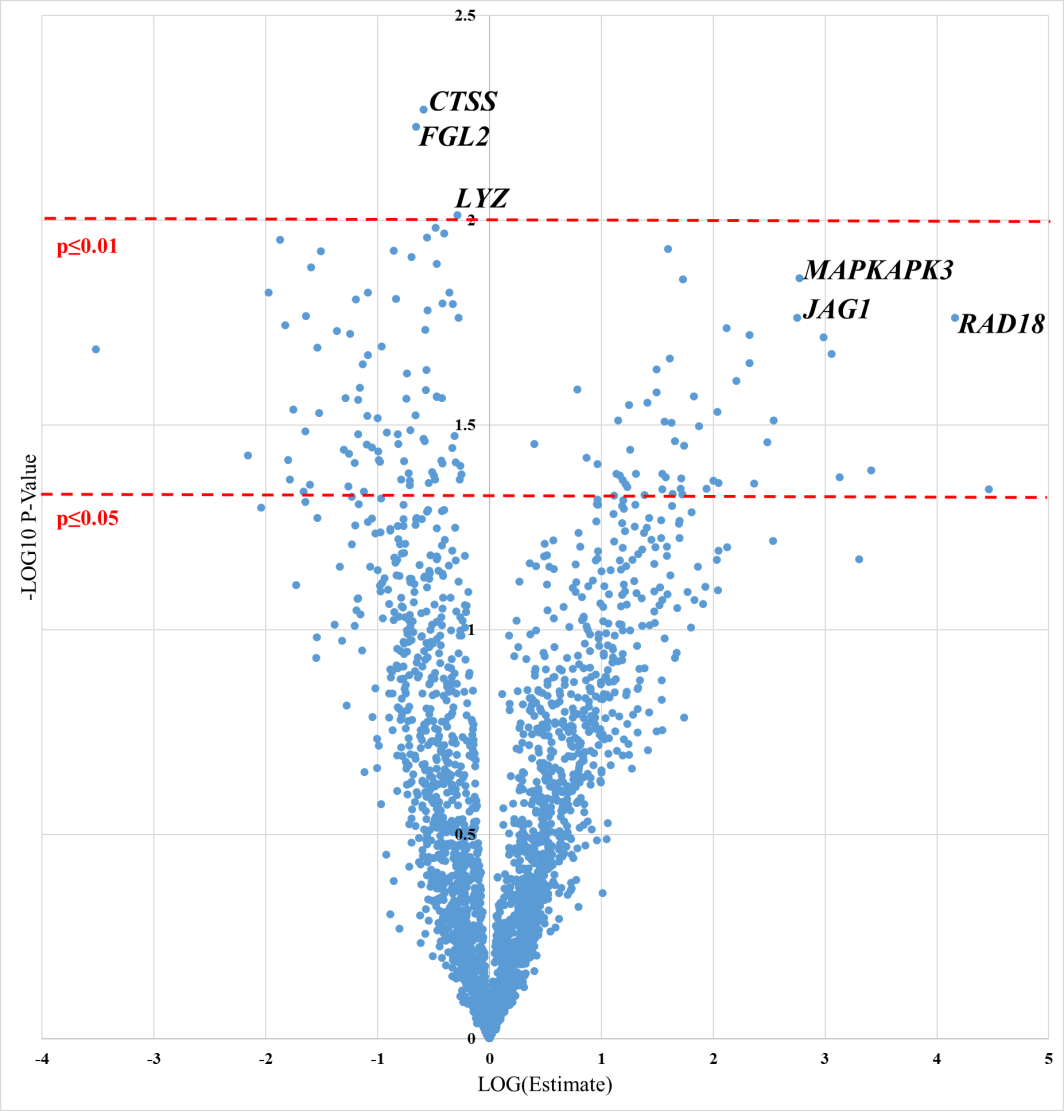
Association between gene expression and bacteremia using a logistic regression model. Top genes are names; nominal significance levels of 95% and 99% are indicated with dashed red line; the x axis represents the strength of the association and the y-axis the significance–genes to the right of the plot are more highly expressed in cases than controls, genes to the left are more highly expressed among the controls.

Evidence of separation in the gene expression profile of cases (n = 7) and controls (n = 22) was also suggested by a PCA model based on 2,560 mRNAs. The PLS-DA model (**Fig 4**) resulted in an R^2^ of 0.60, but a Q^2^ of only 0.09, and again permutation testing indicated that this model was not robust (p = 0.974). Differential expression analysis identified 150 nominally significant genes, of which three were significant at p<0.01 (**Fig 5**). The top genes included *CTSS* (p = 0.005), *FGL2* (p = 0.006), *LYZ* (p = 0.009), *PPBP* (p = 0.010), *CYBB* (p = 0.011) and *CD86* (p = 0.011). The expression levels of the top eight over-expressed genes and the top eight under expressed-genes in cases versus controls are shown in **Figure D in S1 File.** G.profiler analysis determined that the significant genes were enriched for 24 biological terms, including a number relating to vesicle mediated transport, and cytokines (**Table B in S1 File**). When LASSO regression was employed, only three genes were retained in the model: *RAD18*, which encodes a DNA repair protein, *MAPKAPK3*, a kinase activated in response to cellular stress and *JAG1*, which has a reported role in hematopoiesis. Sensitivity analyses were not performed on the gene-expression data due to sample size limitations.

**Fig 6:**
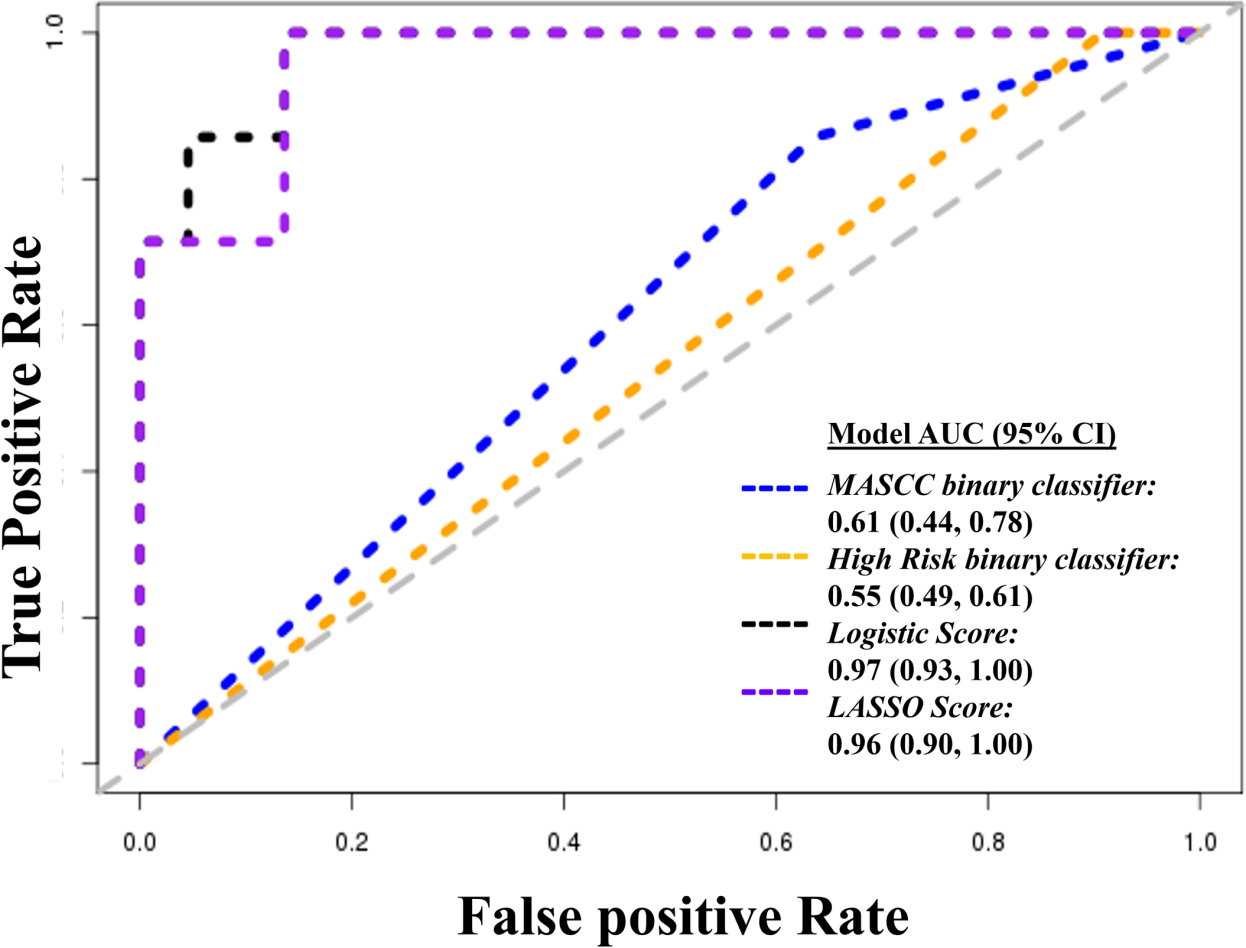
Receiver operating characteristic curves showing the performance of gene expression based (logistic and LASSO) predictors compared to existing clinical (MASCC and high-risk) classifiers. AUC–Area under the receiver operating characteristic curve. MASCC binary classifier–Multinational Association for Supportive Care in Cancer score categorized into <21 (high risk) and ≥21(low risk). High risk binary classifier–defines a patient as high risk if the MASCC score is <21 or if any of the Infectious Diseases Society of America/American Society of Clinical Oncology high risk criteria are met. Logistic score–summary score based on the 153 genes associated with bacteremia under a logistic regression model. LASSO score–summary score based on the 2 genes associated with bacteremia under a penalized LASSO model.

In this subset of patients the AUC for the MASCC classifier was 0.610 (95%CI: 0.437–0.784) and for the high-risk classifier0.546 (95% CI: 0.484–0.607). Again, the omics-based scores outperformed these metrics. The summary score based on the 150 genes had an AUC of 0.974 (95%: 0.923–1.000) (*p dif* MASCC classifier = 1.21x10^-4^; *p dif* high risk classifier<2.20x10^-16^), while the AUC for a model including *RAD18* (p in logistic model = 0.017), *MAPKAPK3* (p in logistic model = 0.014) and *JAG1* (p in logistic model = 0.017) as predictors was 0.961 (95%CI: 0.896 1.000) (*p dif* MASCC classifier = 5.51x10^-6^; *p dif* high risk classifier <2.20x10^-16^) (**Fig 6**). The binary MASCC and high-risk classifiers in this gene expression subpopulation were sensitive (89% and 100%, respectively), but had low specificities (36% and 9%). In contrast, with 100% sensitivity, the specificities was 86% for both our standard logistic and LASSO genomic classifiers (**Table 2**).

### Integrative analysis

When we combined the metabolomic and gene expression data into a single data set, there were too few observations to demonstrate predictive ability, because we had both datasets for only 28 participants. This caused the number of parameters to exceed the number of observations by too wide a margin for our models to converge on a solution. However, integrative analysis using IMPaLA revealed that a number of the genes and metabolites independently identified as significant in the previous analyses are involved in the same biological pathways and processes (**Table 3**). Forty eight pathways were identified as being differentially perturbed in terms of both metabolomics and gene expression (joint q-value<0.05). These included pathways relating to the immune system (joint q-value = 6.29x10^-8^) insulin regulation (including the Insulin receptor signaling cascade; joint q-value = 6.29x10^-8^, IRS-related events triggered by IGF1R;joint q-value = 4.33x10^-3^), the MAPK signaling pathway (joint q-value = 8.38x10^-3^) and multiple pathways involved in the signaling processes necessary for cell survival, differentiation and apoptosis, (such as signaling by NGF; joint q-value = 3.73x10^-45^, signaling by EGFR; joint q-value = 7.25x10^-4^ andDAP12 signaling; joint q-value = 4.49x10^-3^).

**Table 3:** Pathways differentially enriched at metabolomic and transcriptomic levels.

Process	# Overlapping Genes	Genetic p-value	Genetic q-value	# Overlapping metabolites	Metabolomic p-value	Metabolomic q-value	Joint p-value	Joint q-value
Immune System	52	1.28E-10	5.51E-07	5	8.39E-03	3.52E-01	3.06E-11	6.29E-08
Innate Immune System	37	3.36E-08	2.90E-05	3	1.08E-01	8.46E-01	7.40E-08	7.61E-05
Signaling by NGF	16	1.90E-05	3.53E-03	4	1.56E-03	2.01E-01	5.44E-07	3.73E-04
Cytokine Signaling in Immune system	20	3.36E-07	1.45E-04	1	2.83E-01	1.00E+00	1.64E-06	6.73E-04
Signaling by EGFR	15	2.12E-06	7.64E-04	2	6.94E-02	7.59E-01	2.47E-06	7.25E-04
PI3K-Akt signaling pathway—Homo sapiens (human)	15	4.82E-06	1.39E-03	1	6.43E-02	7.26E-01	4.95E-06	1.27E-03
HIF-1 signaling pathway—Homo sapiens (human)	7	1.37E-04	9.67E-03	3	3.73E-03	3.16E-01	7.88E-06	1.80E-03
Transmembrane transport of small molecules	7	5.36E-01	1.00E+00	15	1.20E-06	4.80E-03	9.84E-06	1.97E-03
Chemokine signaling pathway—Homo sapiens (human)	11	6.61E-06	1.72E-03	1	1.05E-01	8.26E-01	1.05E-05	1.97E-03
Insulin receptor signaling cascade	12	2.05E-05	3.53E-03	2	4.11E-02	6.14E-01	1.26E-05	2.16E-03
Integrated Breast Cancer Pathway	6	8.33E-05	7.26E-03	2	1.53E-02	4.04E-01	1.86E-05	2.83E-03
Adaptive Immune System	20	4.70E-04	2.23E-02	4	2.82E-03	2.55E-01	1.92E-05	2.83E-03
NGF signaling via TRKA from the plasma membrane	14	2.14E-05	3.55E-03	2	7.57E-02	7.59E-01	2.32E-05	3.18E-03
Signal Transduction	45	3.49E-04	1.80E-02	10	5.30E-03	3.24E-01	2.63E-05	3.38E-03
Signaling by Insulin receptor	12	5.19E-05	6.77E-03	2	4.11E-02	6.14E-01	3.00E-05	3.42E-03
Downstream signal transduction	13	3.18E-05	4.91E-03	2	6.33E-02	7.26E-01	2.84E-05	3.42E-03
DAP12 signaling	13	3.64E-05	5.42E-03	2	6.33E-02	7.26E-01	3.22E-05	3.49E-03
Central carbon metabolism in cancer—Homo sapiens (human)	6	9.07E-05	7.26E-03	3	4.24E-02	6.14E-01	5.18E-05	4.33E-03
IRS-mediated signaling	11	9.00E-05	7.26E-03	2	4.11E-02	6.14E-01	5.00E-05	4.33E-03
IRS-related events triggered by IGF1R	11	1.04E-04	8.01E-03	2	4.11E-02	6.14E-01	5.70E-05	4.33E-03
IGF1R signaling cascade	11	1.04E-04	8.01E-03	2	4.11E-02	6.14E-01	5.70E-05	4.33E-03
Signaling by Type 1 Insulin-like Growth Factor 1 Receptor (IGF1R)	11	1.08E-04	8.01E-03	2	4.11E-02	6.14E-01	5.89E-05	4.33E-03
DAP12 interactions	13	5.92E-05	6.91E-03	2	6.33E-02	7.26E-01	5.06E-05	4.33E-03
Signaling by PDGF	13	6.91E-05	7.18E-03	2	6.33E-02	7.26E-01	5.84E-05	4.33E-03
GPCR signaling-G alpha s PKA and ERK	12	7.06E-05	7.18E-03	1	8.48E-02	7.59E-01	7.80E-05	5.53E-03
Diseases of signal transduction	12	2.05E-05	3.53E-03	1	3.30E-01	1.00E+00	8.70E-05	5.81E-03
Chemokine signaling pathway	9	8.44E-05	7.26E-03	1	1.05E-01	8.26E-01	1.12E-04	7.18E-03
MAPK Signaling Pathway	8	5.27E-04	2.42E-02	1	2.19E-02	4.38E-01	1.43E-04	8.38E-03
Pathways in cancer—Homo sapiens (human)	14	1.18E-04	8.55E-03	2	9.55E-02	8.26E-01	1.40E-04	8.38E-03
UMP Synthase Deficiency (Orotic Aciduria)	3	1.96E-03	5.47E-02	5	7.26E-03	3.29E-01	1.73E-04	8.66E-03
Pyrimidine Metabolism	3	1.96E-03	5.47E-02	5	7.26E-03	3.29E-01	1.73E-04	8.66E-03
MNGIE (Mitochondrial Neurogastrointestinal Encephalopathy)	3	1.96E-03	5.47E-02	5	7.26E-03	3.29E-01	1.73E-04	8.66E-03
Dihydropyrimidinase Deficiency	3	1.96E-03	5.47E-02	5	7.26E-03	3.29E-01	1.73E-04	8.66E-03
Beta Ureidopropionase Deficiency	3	1.96E-03	5.47E-02	5	7.26E-03	3.29E-01	1.73E-04	8.66E-03
GPCR signaling-cholera toxin	11	1.90E-04	1.22E-02	1	8.48E-02	7.59E-01	1.94E-04	9.51E-03
Ca-dependent events	1	2.82E-01	8.54E-01	4	5.98E-05	5.40E-02	2.02E-04	9.68E-03
Pyrimidine nucleotides nucleosides metabolism	3	1.94E-02	2.20E-01	6	9.34E-04	1.49E-01	2.16E-04	9.86E-03
Ascorbate and aldarate metabolism—Homo sapiens (human)	1	2.58E-01	8.12E-01	5	8.12E-05	5.40E-02	2.47E-04	1.08E-02
Metabolism	19	8.04E-01	1.00E+00	32	3.36E-05	4.46E-02	3.11E-04	1.33E-02
PLC beta mediated events	2	9.41E-02	4.94E-01	4	3.91E-04	1.20E-01	4.12E-04	1.73E-02
G-protein mediated events	2	9.75E-02	5.04E-01	4	3.91E-04	1.20E-01	4.26E-04	1.75E-02
Hemostasis	20	6.59E-05	7.18E-03	1	6.17E-01	1.00E+00	4.52E-04	1.82E-02
Disease	15	5.10E-04	2.37E-02	3	9.87E-02	8.26E-01	5.48E-04	2.09E-02
HTLV-I infection—Homo sapiens (human)	10	5.54E-04	2.47E-02	1	1.05E-01	8.26E-01	6.24E-04	2.29E-02
GPCR signaling-G alpha s Epac and ERK	10	8.33E-04	3.30E-02	1	8.48E-02	7.59E-01	7.45E-04	2.60E-02
SLC-mediated transmembrane transport	4	3.82E-01	1.00E+00	11	1.98E-04	8.75E-02	7.92E-04	2.67E-02
Pyrimidine catabolism	1	1.24E-01	5.67E-01	5	9.23E-04	1.49E-01	1.15E-03	3.54E-02
PKB-mediated events	3	9.52E-03	1.41E-01	2	1.53E-02	4.04E-01	1.43E-03	4.21E-02

Overlapping–indicates gene/metabolite are found both in the named pathway and among the set identified as significantly associated with bacteremia in this population

Enrichment p and q values are provided for the metabolites alone, the genes alone and the joint enrichment

Q-value–p-value adjusted for the false discovery rate

## Discussion

We have demonstrated methods for discovery of a multi-omics-based predictor for detection of bacteremia among FN patients without apparent infection. Omic profiles are increasingly being leveraged as predictive, diagnostic and prognostic biomarkers, and can provide insight into the underlying biology.[32, 33] Metabolomic and transcriptomic diagnostics have already been deployed clinically. For example, real-time analysis of the metabolome is a clinical reality, as exemplified by the iKnife, which performs instantaneous analysis of the mucosal lipidome to phenotype colorectal cancer during surgery.[34] Similarly, transcriptomics have yielded diagnostic tests that are approaching clinical implementation for detection of serious bacterial infection.[35, 36] To our knowledge, this was the first study to develop multi-omic based biomarkers of bacteremic FN; one prior study used metabolomics alone to investigate infection in the setting of FN.[37] Our future work will apply these methods to a larger sample size, separated into derivation and validation sets, with the goal of developing a clinically-applicable tool for detection of bacteremia during the initial evaluation.

For detection of bacteremia, the two existing clinical predictors; MASCC and the high-risk classifier, were sensitive but demonstrated poor specificity. In contrast, although derived from only a small population, our metabolomic and transcriptomic predictors maintained impressive specificities with 100% sensitivity. These results demonstrate that derivation of omics-based predictors of bacteremia in the setting of FN is feasible, and justify further research in a larger sample.

We found that pregnenolone steroids, which are cortisol precursors, were upregulated in cases relative to controls. Prior research has linked sepsis to an overexpression of cortisol precursors despite normal cortisol levels, implying decreased activity of 3-beta-hydroxysteroid dehydrogenase.[38] Carnitines were down-regulated in cases, which is in agreement with previous evidence for a differential abundance of carnitines in bacteremic compared to non-bacteremic patients.[39, 40] L-carnitine has entered clinical trials as a therapeutic agent for patients with sepsis, and metabolomic analysis has been used to identify the subset of patients responding.[41] Other pathways involved in amino acid metabolism were also perturbed, which could relate to the enhanced extraction of amino acids by the liver noted in patients with sepsis and systemic inflammatory response syndrome.[42] For example, pyrimidine metabolism was differentially regulated, possibly due to the *de novo* synthesis of pyrimidines required for successful proliferation of pathogens in blood.[43] Furthermore, the dysregulation of the ascorbate and aldarate metabolism pathway may relate to the lower circulating levels of ascorbate reported in patients with sepsis.[44]

A number of genes showed differential expression between cases and controls. These included, *PPBP*, an antimicrobial protein with bactericidal and antifungal activity; *CYBB*, which is essential for the microbicidal oxidase system of phagocytes; *LYZ*, which encodes a component of the innate immune system that cleaves peptidoglycan bonds in the bacterial cell wall;,and *CD86* and *FGL2* which have been associated with severity and worse outcomes in sepsis[45, 46] These results suggest that a weaker innate immune response might predispose to bacteremia after depletion of the adaptive immune system by chemotherapy. On a pathway level, the differentially-expressed genes were enriched for a number biological processes including some relating to the release of cytokines, supporting the idea of a unique immunologic milieu in FN patients with bacteremia.

Integrative analysis supported the findings of the individual omics analysis, and provided mechanistic links between the metabolites and genes independently associated with bacteremia. For example, MAPK signaling pathways were perturbed. MAPKs play an important role in the cascade of cellular responses evoked by extracellular stimuli such as pro-inflammatory cytokines or physical stress, and they have been shown to be activated in the setting of bacterial challenge.[47] These integrative results should be viewed with caution due to the limited sample size, which was constrained by funding. However they do provide a good indication of potential gene-metabolite relationships, and a demonstration that in a larger sample, dysregulation at multiple omic levels can be explored simultaneously to interrogate underlying mechanisms.

Regarding limitations, we caution the reader that the sample size, particularly for the transcriptomic data was limited, and we were unable to stratify by potential effect modifiers such as sex.These results should therefore be considered illustrative, and the specific biological entities identified as differentially abundant should be considered exploratory.RNAseq could not be used to quantify expression of the entire transcriptome, because leukocyte-poor blood has insufficient cells for this technique. Therefore, we used EdgeSeq, which does not require an RNA isolation step, but quantifies relative abundance of only 2,560 transcripts. However, the genes included in the EdgeSeq panel were selected on the basis of their relevance to both cancer and the immune system, making them ideally suited to this study population. Because participants had FN as an unscheduled emergency, their dietary intake prior to presenting in the Emergency room may have influenced their metabolome, but this represents the scenario in which a predictor of bacteremia would ultimately be utilized. However, we were able to provide evidence that prior antibiotic use was not influencing our results; nor was the type or site of the initial tumor. Furthermore, within the limited sample size we were able to demonstrate that the metabolomic profile appeared to be similar for Gram-negative and Gram-positive bacteremia,although it would be of interest to explore this further in the larger sample.

In conclusion, we generated metabolomic and transcriptomic predictors of bacteremia, among FN patients without apparent infection at presentation. With overfitting as a caveat, these predictors significantly outperformed currently-recommended risk-stratification tools, with markedly improved specificity and perfect sensitivity. Interrogation of differentially-abundant biomarkers revealed biologically-plausible roles in bacteremia within the setting of FN. This is the first such study within a field that is in dire need of novel biomarkers and management strategies. Via further study in a larger sample with discovery and validation sets, development of a biologically meaningful objective predictor that utilizes both clinical and omics data is feasible, and will facilitate early diagnosis. This, in turn, will enable appropriate aggressive treatment for patients predicted to have bacteremia, and appropriate conservative treatment of those predicted not to have bacteremia.

## Supporting information

S1 Methods Supplementary methods. (DOCX)

S1 File **Table A: Metabolites significantly (p≤0.01) associated with bacteremia.** *Effect estimates adjusted for age*, *sex*, *BMI and tumor type (liquid or solid)*. *Metabolites with an X- prefix are awaiting annotation*. **Table B; Biological processes and pathways enriched among 150 genes significantly associated with bacteremia.** *Enrichment analysis performed using the g*.*GOSt tools from the g*.*profiler package (**http*:*//biit*.*cs*.*ut*.*ee/gprofiler/**) p-values are Bonferonni corrected*. **Figure A: Metabolomic PCA by Case-Control Status. Figure B: Metabolomic PCA by Other Clinical Variables. Figure C: Relative Metabolite Intensity levels in Bacteremia Cases and Controls for the Top Eight Upregulated and Top Eight Downregulated Metabolites. Figure D: Relative Metabolite Intensity levels in Bacteremia Cases stratified by Gram status (negative or positive) and Controls (CO) for the Top Eight Upregulated and Top Eight Downregulated Metabolites. Figure E: Expression levels in Bacteremia Cases and Controls for the Top Eight Overexpressed and Top Eight Unexpressed Genes.**
(DOCX)

S1 Dataset Gene expression data/metabolomics data and the relevant epidemiological and clinical variables for 29 subjects. (CSV)

S2 Dataset Metabolomics data and the relevant epidemiological and clinical variables for 39 subjects. (CSV)

S3 Dataset Biochemical names of the metabolites profiled. (CSV)
