# Supplementary material for: Integrative omics to detect bacteremia in patients with febrile neutropenia
Source: PLoS One. 2018 May 16;13(5):e0197049. doi: 10.1371/journal.pone.0197049 (PMC5955575; doi:10.1371/journal.pone.0197049)
Supplement: S1 Methods — (DOCX) [file pone.0197049.s001.docx]

**Integrative omics to detect Bacteremia in Patients with Febrile Neutropenia:**

**Supplementary methods**

**Metabolomic profiling**

Samples were collected in lithium-heparin tubes and stored at –70°C until processed. Sample preparation and analysis was performed at Metabolon, Inc (Durham, NC). Briefly, recovery standards were added prior to the first step in the extraction process for quality control purposes, as described previously.^1^ To remove protein, dissociate small molecules bound to protein or trapped in the precipitated protein matrix, and to recover chemically diverse metabolites, proteins were precipitated with methanol under vigorous shaking for two minutes followed by centrifugation. The resulting clarified supernatant extract was divided into five aliquots, one for each of the four individual LC–MS analyses (LC MS Pos Polar, LC MS Pos Lipid, LC MS Polar, LS MS Neg) and one spare, briefly evaporated to remove the organic solvent and stored overnight under nitrogen before preparation for analysis. All of the profiling methods alternated between full-scan MS and data-dependent MS^n^ scans. The scan range varied slightly between methods but generally covered 70–1000 *m*/*z*.

Experimentally detected metabolites were identified by matching the ion chromatographic retention index, accurate mass, and mass spectral fragmentation signatures with a reference library consisting of over 4000 entries created from authentic standard metabolites under the analytical procedures identical to those used for the experimental samples (in-house peak detection and integration software was used.^2^ The data output from the software was a list of *m*/*z* ratios, fragmentation spectra, retention indices, and area under the curve (AUC) values. All proposed identifications were then reviewed and curated by manual oversight to confirm biochemical identification based on the criteria above.^2^

**Gene expression profiling**

EdgeSeq from HTG Molecular, Inc.^3^, was used to quantify the expression of 2,560 mRNA probes. Blood was collected directly into PAXgene tubes and frozen at –80^o^C. Concentration was measured by electrochemiluminescence immunoassay, using a Roche BRAHMS PCT assay run on a Roche Cobas e601 Immunoassay analyzer (Roche Diagnostics, Indianapolis, IN).^4^

References

1. Evans A, Bridgewater B, Liu Q, et al. High Resolution Mass Spectrometry Improves Data Quantity and Quality as Compared to Unit Mass Resolution Mass Spectrometry in HighThroughput Profiling Metabolomics. metabolomics 2014;4.

2. Dehaven CD, Evans AM, Dai H, Lawton KA. Organization of GC/MS and LC/MS metabolomics data into chemical libraries. Journal of cheminformatics 2010;2:9.

3. Girard L, Rodriguez-Canales J, Behrens C, et al. An Expression Signature as an Aid to the Histologic Classification of Non-Small Cell Lung Cancer. Clin Cancer Res 2016;22:4880-9.

4. HTG EdgeSeq Oncology Biomarker Panel 2017. (Accessed 7/13/2017, at <https://www.htgmolecular.com/products/mrna-oncology-biomarkers>.)
