# Supplementary material for: Integrative omics to detect bacteremia in patients with febrile neutropenia
Source: PLoS One. 2018 May 16;13(5):e0197049. doi: 10.1371/journal.pone.0197049 (PMC5955575; doi:10.1371/journal.pone.0197049)
Supplement: S1 File — Table A: Metabolites significantly (p≤0.01) associated with bacteremia. Effect estimates adjusted for age, sex, BMI and tumor type (liquid or solid). Metabolites with an X- prefix are awaiting annotation. Table B; Biological processes and pathways enriched among 150 genes significantly associated with bacteremia. Enrichment analysis performed using the g.GOSt tools from the g.profiler package (http://biit.cs.ut.ee/gprofiler/) p-values are Bonferonni corrected. Figure A: Metabolomic PCA by Case-Control Status. Figure B: Metabolomic PCA by Other Clinical Variables. Figure C: Relative Metabolite Intensity levels in Bacteremia Cases and Controls for the Top Eight Upregulated and Top Eight Downregulated Metabolites. Figure D: Relative Metabolite Intensity levels in Bacteremia Cases stratified by Gram status (negative or positive) and Controls (CO) for the Top Eight Upregulated and Top Eight Downregulated Metabolites. Figure E: Expression levels in Bacteremia Cases and Controls for the Top Eight Overexpressed and Top Eight Unexpressed Genes. (DOCX) [file pone.0197049.s002.docx]

**Integrative omics to detect Bacteremia in Patients with Febrile Neutropenia:**

**Table A**

| **Metabolite** | **Super-pathway** | **Sub-pathway** | **HMDB ID** | **OR** | **95%CI** | **P** |
| --- | --- | --- | --- | --- | --- | --- |
| 17alpha-hydroxypregnanolone glucuronide | Lipid | Pregnenolone Steroids |  | 18.60 | (3.65,302.51) | 5.1x10^-3^ |
| arachidoylcarnitine (C20)* | Lipid | Fatty Acid Metabolism(Acyl Carnitine) | HMDB06460 | 0.03 | (1.4x10^-3^,0.25) | 5.7x10^-3^ |
| estrone 3-sulfate | Lipid | Estrogenic Steroids | HMDB01425 | 6.85 | (2.07,36.54) | 6.3x10^-3^ |
| 4-acetamidobutanoate | Amino Acid | Polyamine Metabolism | HMDB03681 | 58.36 | (5.1,2318.54) | 6.4x10^-3^ |
| sphingomyelin (d18:0/20:0, d16:0/22:0)* | Lipid | Sphingolipid Metabolism |  | 0.01 | (2.4x10^-4^,0.18) | 6.9x10^-3^ |
| margaroylcarnitine* | Lipid | Fatty Acid Metabolism(Acyl Carnitine) | HMDB06210 | 0.05 | (3.2x10^-3^,0.31) | 7.1x10^-3^ |
| X - 19561 |  |  |  | 19.08 | (3.23,299.35) | 7.3x10^-3^ |
| stearoylcarnitine (C18) | Lipid | Fatty Acid Metabolism(Acyl Carnitine) | HMDB00848 | 0.01 | (1.6x10^-4^,0.16) | 7.6x10^-3^ |
| ximenoylcarnitine (C26:1)* | Lipid | Fatty Acid Metabolism(Acyl Carnitine) |  | 0.03 | (1.4x10^-3^,0.26) | 7.6x10^-3^ |
| 1-palmitoyl-2-oleoyl-GPC (16:0/18:1) | Lipid | Phosphatidylcholine (PC) | HMDB07972 | 321.54 | (8.75,71361.69) | 8.1x10^-3^ |
| palmitoylcarnitine (C16) | Lipid | Fatty Acid Metabolism(Acyl Carnitine) | HMDB00222 | 0.04 | (2.5x10^-3^,0.31) | 8.2x10^-3^ |
| myristoyl dihydrosphingomyelin (d18:0/14:0)* | Lipid | Sphingolipid Metabolism | HMDB12085 | 0.01 | (8.0x10^-5^,0.17) | 8.4x10^-3^ |
| X - 22515 |  |  |  | 4.50 | (1.64,17.22) | 8.6x10^-3^ |
| N-acetylputrescine | Amino Acid | Polyamine Metabolism | HMDB02064 | 45.16 | (4.42,1632.01) | 8.8x10^-3^ |
| X - 12117 |  |  |  | 28.92 | (3.75,771.97) | 9.2x10^-3^ |
| myristoylcarnitine (C14) | Lipid | Fatty Acid Metabolism(Acyl Carnitine) | HMDB05066 | 0.06 | (4.1x10^-3^,0.36) | 9.3x10^-3^ |
| dihomo-linolenoylcarnitine (20:3n3 or 6)* | Lipid | Fatty Acid Metabolism(Acyl Carnitine) |  | 0.06 | (3.9x10^-3^,0.35) | 9.5x10^-3^ |
| maleate | Lipid | Fatty Acid, Dicarboxylate | HMDB00176 | 39.25 | (4.02,1430.14) | 9.9x10^-3^ |
| X - 24334 |  |  |  | 82.08 | (6.1,7720) | 1.0x10^-2^ |

**Table B**

| **GO Term** | **# Genes in set** | **# Bactermia genes in Set** | **p-value** |
| --- | --- | --- | --- |
| response to stimulus | 7030 | 105 | 1.10x10^-11^ |
| receptor binding | 1254 | 36 | 7.24x10^-8^ |
| Cytokine Signaling in Immune system | 826 | 32 | 1.89x10^-7^ |
| protein kinase activity | 570 | 21 | 2.43x10^-5^ |
| endocytic vesicle | 233 | 13 | 1.71x10^-4^ |
| Cytokine-cytokine receptor interaction | 268 | 16 | 3.04x10^-4^ |
| plasma membrane part | 2179 | 41 | 6.29x10^-4^ |
| Transcriptional misregulation in cancer | 184 | 12 | 0.002 |
| molecular transducer activity | 1402 | 30 | 0.003 |
| cell surface | 555 | 17 | 0.009 |
| PI3K-Akt signaling pathway | 351 | 16 | 0.009 |
| cytokine receptor activity | 79 | 7 | 0.009 |
| Chemokine signaling pathway | 183 | 11 | 0.011 |
| regulation of endopeptidase activity | 247 | 11 | 0.016 |
| Ephrin signaling | 20 | 4 | 0.025 |
| Signaling by EGFR | 460 | 16 | 0.025 |
| PI3K/AKT Signaling in Cancer | 90 | 7 | 0.027 |
| ErbB signaling pathway | 86 | 7 | 0.031 |
| Bladder cancer | 41 | 5 | 0.034 |
| leukocyte cell-cell adhesion | 269 | 11 | 0.035 |
| Central carbon metabolism in cancer | 66 | 6 | 0.046 |
| Acute myeloid leukemia | 66 | 6 | 0.046 |
| MAPK family signaling cascades | 292 | 12 | 0.048 |
| Abnormality of macrophages | 27 | 5 | 0.0500 |

**Figure A**


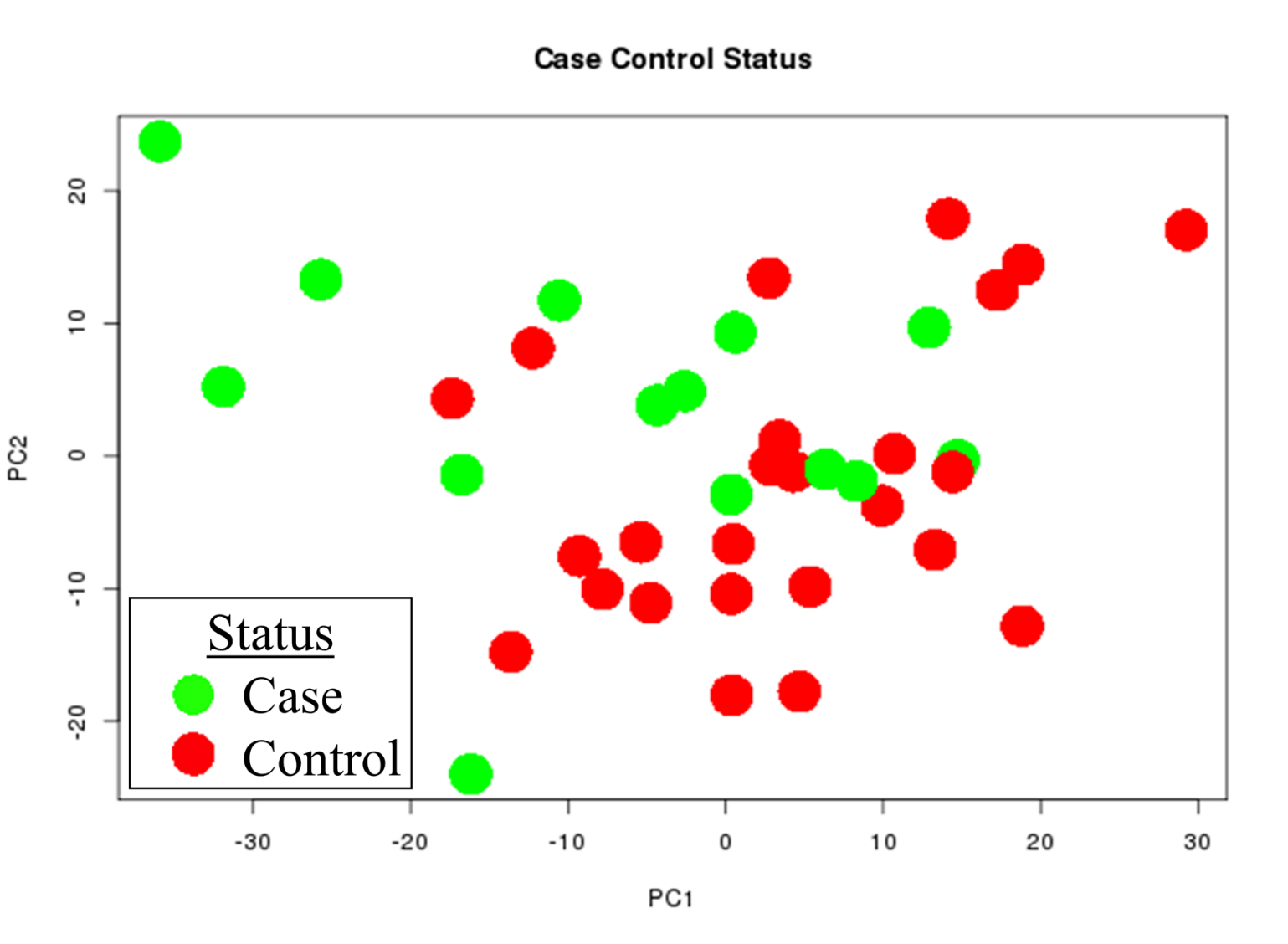


**Figure B**

**
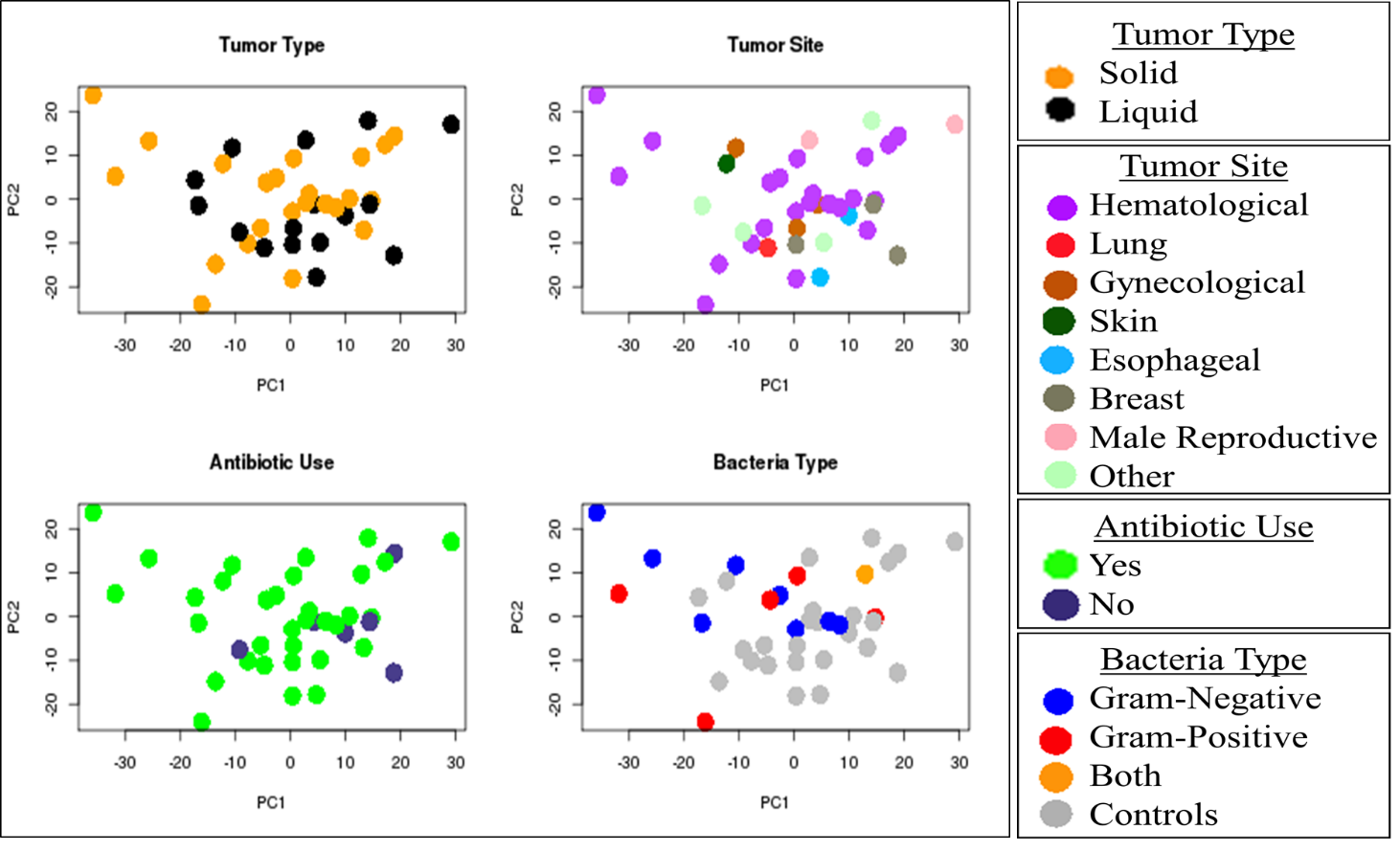
**

**Figure C**

**
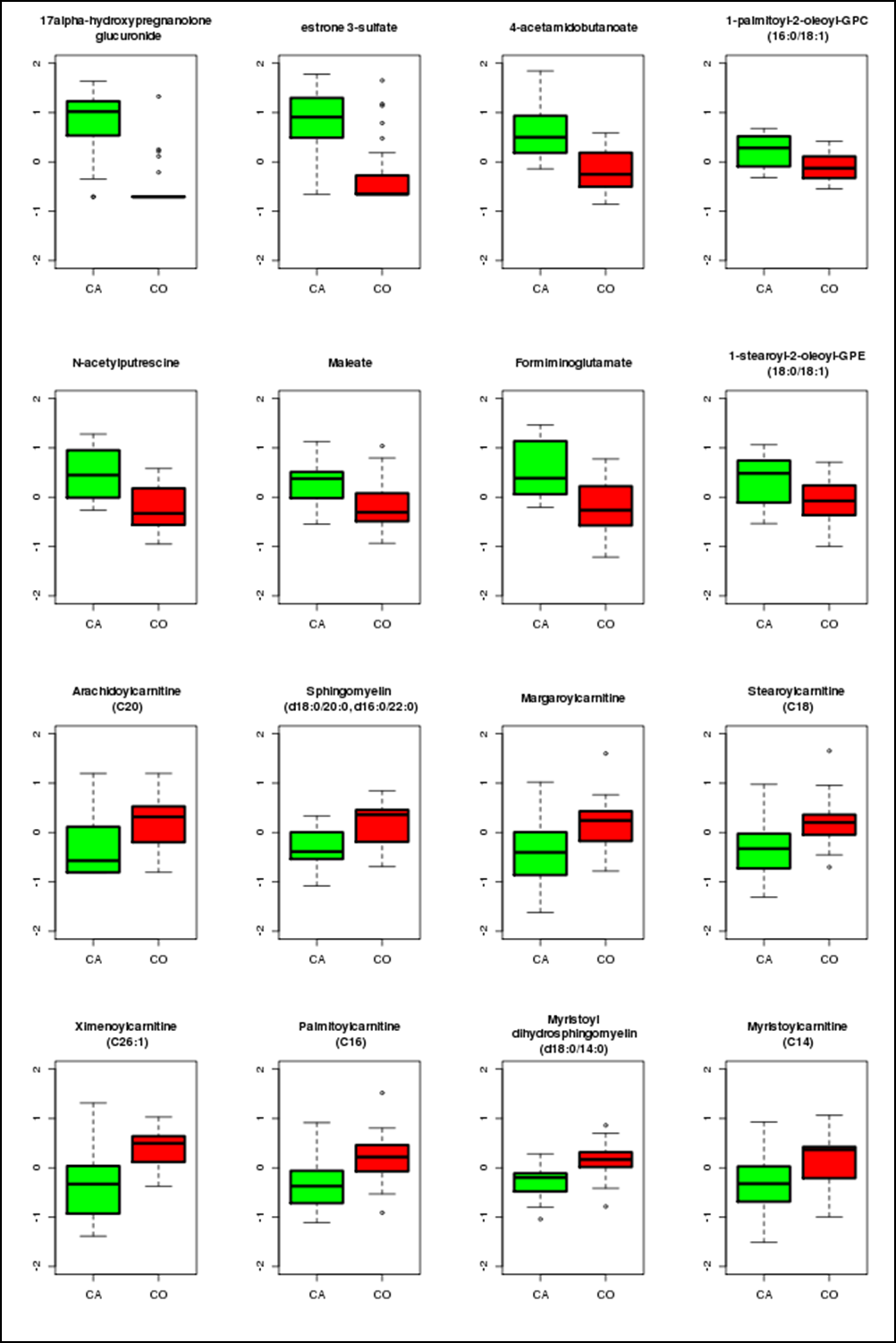
**

**Figure D**

**
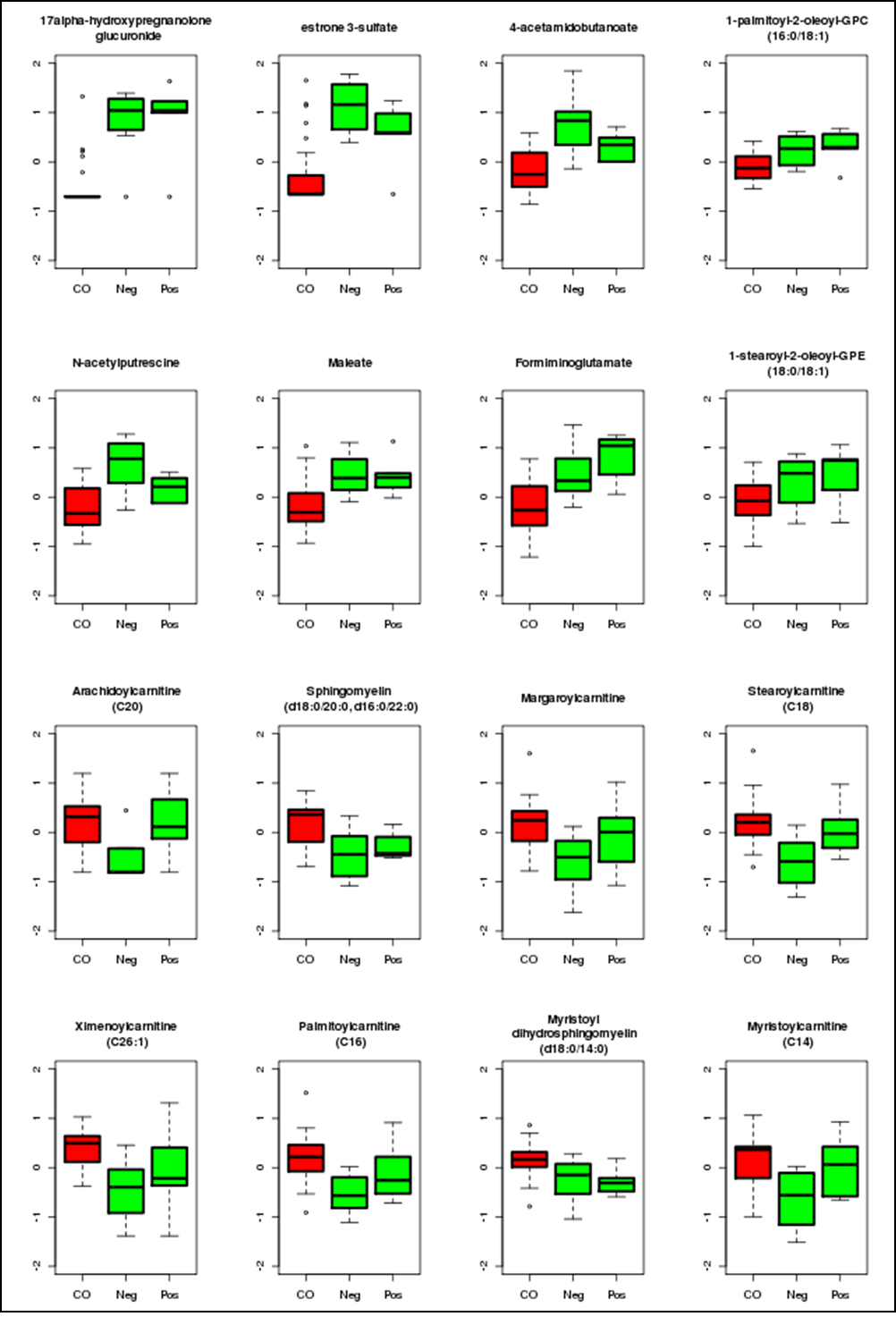
**

**Figure E**

**
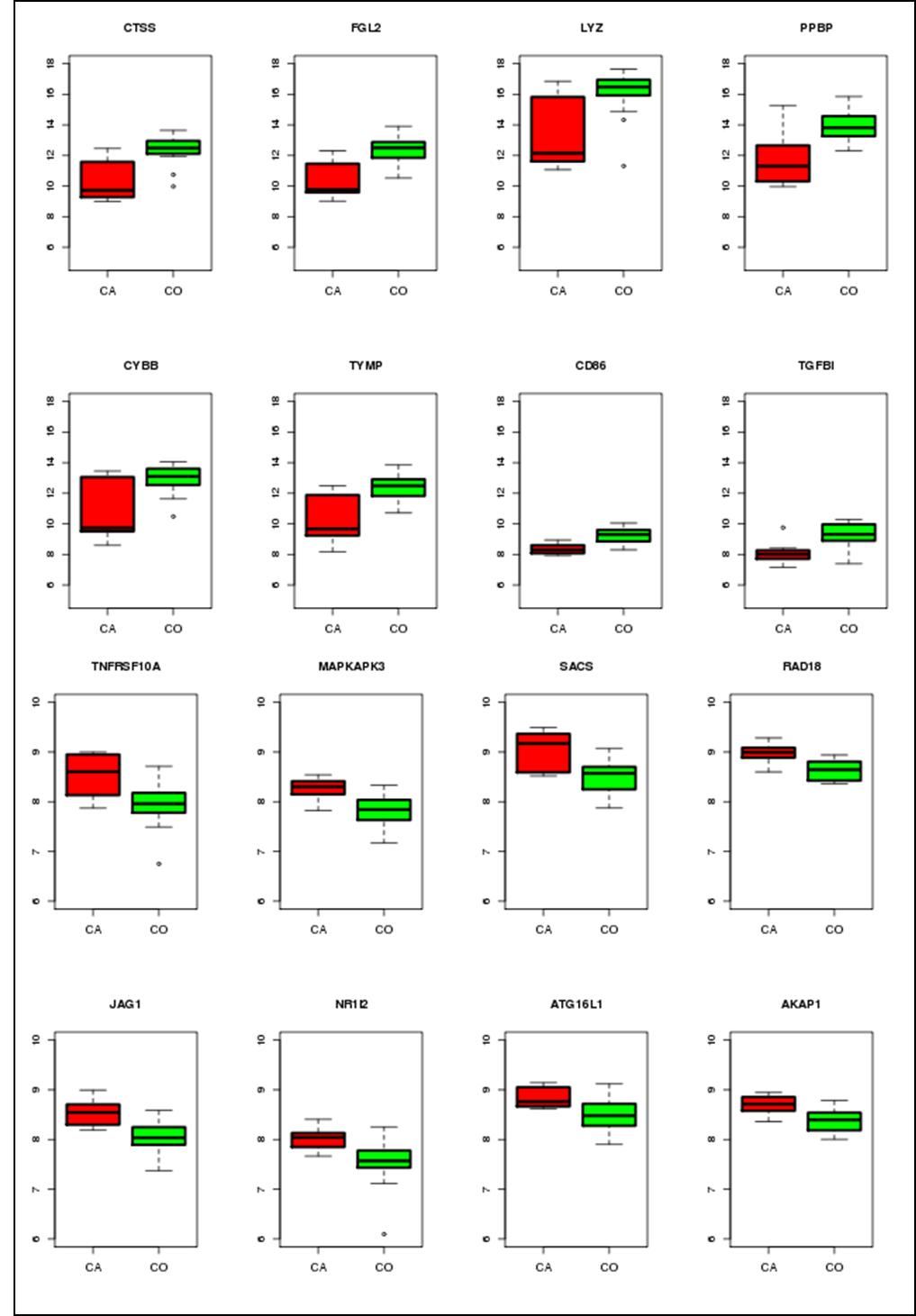
**
